# Variation in Stem Anatomical Characteristics of Campanuloideae Species in Relation to Evolutionary History and Ecological Preferences

**DOI:** 10.1371/journal.pone.0088199

**Published:** 2014-02-21

**Authors:** Fritz Hans Schweingruber, Pavel Říha, Jiří Doležal

**Affiliations:** 1 Swiss Federal Research Institute WSL, Birmensdorf, Switzerland; 2 Section of Plant Ecology, Institute of Botany, Academy of Sciences of the Czech Republic, Třeboň, Czech Republic; 3 Department of Botany, Faculty of Science, University of South Bohemia, České Budějovice, Czech Republic; Institute of Botany, Chinese Academy of Sciences, China

## Abstract

**Background:**

The detailed knowledge of plant anatomical characters and their variation among closely related taxa is key to understanding their evolution and function. We examined anatomical variation in 46 herbaceous taxa from the subfamily Campanuloideae (Campanulaceae) to link this information with their phylogeny, ecology and comparative material of 56 woody tropical taxa from the subfamily Lobelioideae. The species studied covered major environmental gradients from Mediterranean to Arctic zones, allowing us to test hypotheses on the evolution of anatomical structure in relation to plant competitive ability and ecological preferences.

**Methodology/Principal Findings:**

To understand the evolution of anatomical diversity, we reconstructed the phylogeny of studied species from nucleotide sequences and examined the distribution of anatomical characters on the resulting phylogenetic tree. Redundancy analysis, with phylogenetic corrections, was used to separate the evolutionary inertia from the adaptation to the environment. A large anatomical diversity exists within the Campanuloideae. Traits connected with the quality of fibres were the most congruent with phylogeny, and the *Rapunculus* 2 (“phyteumoid”) clade was especially distinguished by a number of characters (absence of fibres, pervasive parenchyma, type of rays) from two other clades (*Campanula* s. str. and *Rapunculus 1*) characterized by the dominance of fibres and the absence of parenchyma. Septate fibres are an exclusive trait in the Lobelioideae, separating it clearly from the Campanuloideae where annual rings, pervasive parenchyma and crystals in the phellem are characteristic features.

**Conclusions/Significance:**

Despite clear phylogenetic inertia in the anatomical features studied, the ecological attributes and plant height had a significant effect on anatomical divergence. From all three evolutionary clades, the taller species converged towards similar anatomical structure, characterized by a smaller number of early wood vessels of large diameter, thinner cell-walls and alternate intervessel pits, while the opposite trend was found in small Arctic and alpine taxa. This supports the existing generalization that narrower vessels allow plants to grow in colder places where they can avoid freezing-induced embolism, while taller plants have wider vessels to minimize hydraulic resistance with their greater path lengths.

## Introduction

The detailed knowledge of plant anatomical characters and their variation among closely related taxa is key to understanding their evolution and function [1]. Variation in anatomical structure is a result of several forces such as: the adaptation of species to the prevailing conditions in their habitats [2], phenotypic plasticity as an ability of individuals with an identical genotype to develop differently - based on specific conditions during their ontogeny [3], [4], and evolutionary constraints (phylogenetic inertia) in which taxa that share part of their evolutionary history possess similar ‘blue-prints’ [5]–[7]. One of the critical features of comparative studies on plant trait variations in relation to ecological adaptations is therefore the extent of phylogenetic relatedness among taxa [8], which makes them partly dependent in any statistical inference. In other words, part of the explanatory power uncovered by relating the anatomical traits to ecological preferences might be alternatively explained by phylogenetic inertia affecting both the similarity of anatomical traits among closely related taxa and the similarity of ecological niches that such taxa occupy [9].

Understanding the evolution of plant structures requires separation of evolutionary inertia from a true adaptation to the environment. This is commonly done by comparing analyses made with and without phylogenetic corrections [10]. This approach is based on discounting all of the variation that could possibly be explained by phylogenetic relatedness of the studied species [9], before studying the effect of other potential predictors such as the species’ ecological preferences. The phylogenetic relatedness is presented by a cladogram, the quality of which can affect inferences about adaptations. For instance, phylogenetic trees derived from DNA sequences give more accurate information than traditional taxonomy based on morphological data. The phylogenetic tree is then turned into a distance matrix, representing the distance of any pair of taxa measured along the branches of the tree; this value represents the distance to the nearest common ancestor of the two taxa being compared. The distance matrix is then used as a set of descriptors which can be used as covariates in analyses that need to account for the effect of phylogenetic relatedness first, before focusing on the effect of ecological variables [7], [11].

In this paper, we aim to provide new insights into anatomical stem variation in closely related plant taxa from the subfamily Campanuloideae (Campanulaceae family, belonging to the order of Asterales) and to link this information with their phylogeny and ecology. The Campanulaceae family includes 84 genera with 2400 species [12]. In Europe there are 14 genera representing 209 endemic species [13], with the genera *Campanula* (142 species) and *Phyteuma* (24 species) being most common. Eddie et al. [14] characterized the morphological and phylogenetic features of this family, with representatives of the Campanuloideae subfamily mostly concentrated in the Northern Hemisphere and widely distributed from subtropical Mediterranean to temperate and alpine-Arctic regions. Target species of this study included common taxa from all these habitats, allowing us to test several hypotheses on the evolution of plant structure and function. In general, variations in plant construction should lead to differences in plant physiological function. These differences in morphological structure and physiological function should allow differential tolerance to changes in environmental settings. For instance, in colder places smaller vessels have repeatedly evolved to enable plants to cope with freezing-induced embolism and cavitation [15]. Xylem cavitation diminishes a plant’s capacity to transport water from the soil to the leaves. This reduction in xylem hydraulic conductivity can impair the carbon fixation rate by inducing stomatal closure to prevent further cavitation and desiccation of leaf tissues. In less hostile environments, taller plants should have larger vessels which will, in part, minimize hydraulic resistance by their greater path lengths [2], [16]. The evolutionary and ecological implications of anatomical character variation in different environments have mostly been studied in conifers and deciduous broadleaved trees [17]–[19], with herbaceous plants remaining somewhat neglected.

Very few studies exist on the anatomy of Campanulaceae stems: Metcalfe and Chalk [20] studied two European herbaceous species (*Campanula pyramidalis*, *Asyreunema limonifolium*) of the subfamily Campanuloideae; Carlquist [1] described 56 species of Lobelioideae (summarized by Lammers [12]). Shulkina et al. [21] related anatomical structures of 15 Russian Campanuloideae species to life forms and evolution and Schweingruber et al. [22] made an anatomical survey of 36 stems from Campanuloideae species.

The present study has four goals: a) Describing the stem-anatomical structures of herbaceous plants from seasonal climates within the subfamily Campanuloideae, b) Constructing a phylogenetic tree for target species from nucleotide sequences obtained from genBank, c) Relating stem anatomical features to phylogeny and ecology, and d) Comparing these specimens with the anatomy of shrubs and trees of the tropical subfamily Lobelioideae described by Carlquist [1].

## Materials and Methods

### Target Species

**Figure 1:**
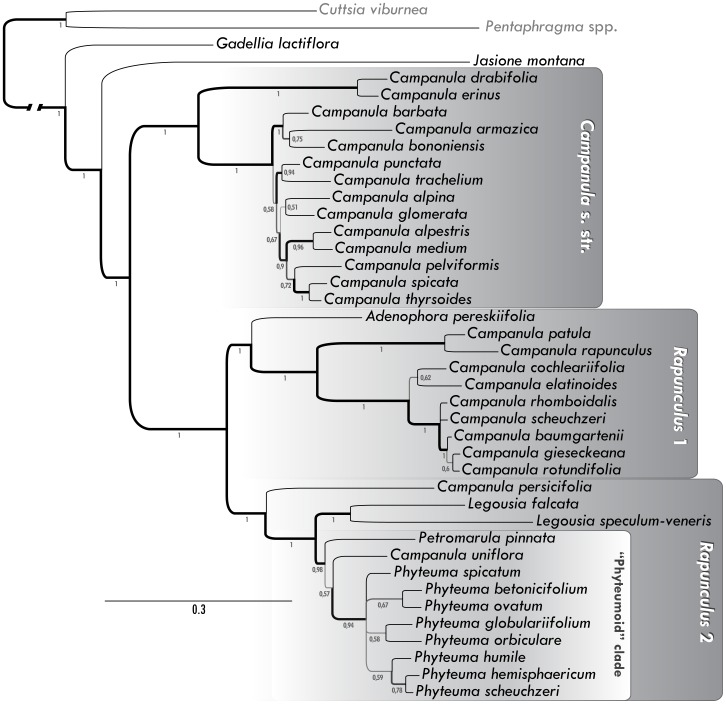
Majority rule consensus of trees sampled by the Bayesian analysis of a concatenated partitioned dataset consisting of ITS, *trnL-trnT* spacer, *matK*, *rbcL* and *petB-petD* spacer sequences. Numbers below the branches indicate Bayesian posterior probability (BPP) values. For better orientation, major clades mentioned in the text are delimited by grey boxes.

The 46 species analyzed in this study were recently collected from their native habitats, mainly in Western Europe. Forty common and relatively widespread species were collected in the Alps and southern Europe, three in Georgia, two in Greenland and one in China along altitudinal gradients ranging from 20 to 3000 m a.s.l. The species mainly represent the European plants (Figure 1) from the Mediterranean zone and within an alpine altitudinal transect north and south of the Alps. Plant size varied between 5 cm and 100 cm. The detailed information on the species studied can be found in Table S1, available online. We classified each species into one of the eight habitat categories representing species growth optima: Arctic zone, alpine meadows, alpine rocks (e.g. screes, rocks, outcrops), low-elevation meadows, low-elevation rock habitats, deciduous mixed forests and the Mediterranean zone. Anatomical codes and photographs of most of the species can be found in an online database (http://www.wsl.ch/dendro/xylemdb/index.php). Plant identification was based on the following references: Europe [13], [23], [24], China [25], Greenland [26], and Georgia [27]. No specific permits were required for the described field collections, the locations were not privately-owned or protected in any way and the field studies did not involve endangered or protected species.

### Anatomical Sections

**Figure 2:**
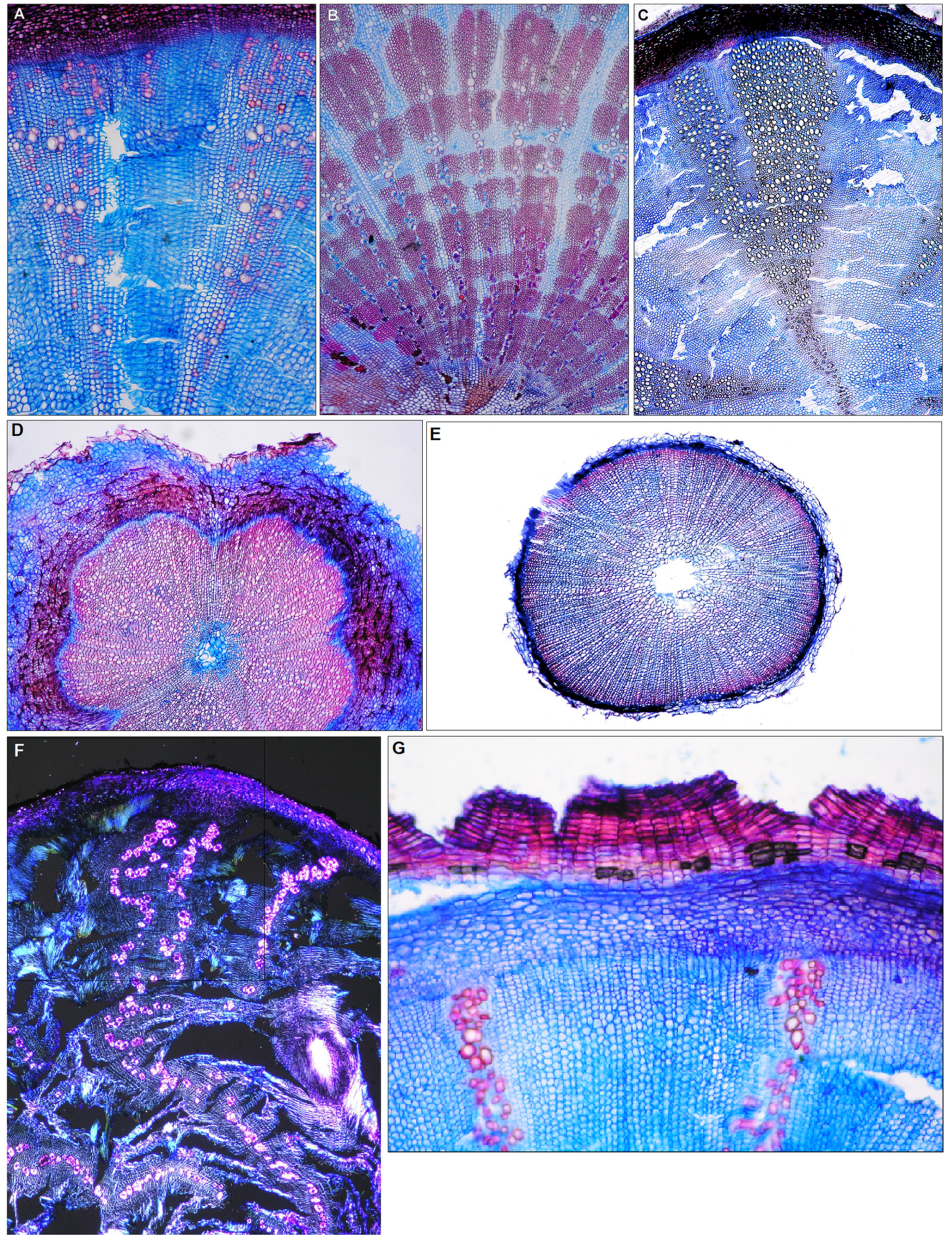
Annual ring boundaries and ray-like structures in herbaceous Camapanulaceae. (A) Semi-ring porous xylem without fibres in root collar of *Campanula rotundifolia*, 20 cm tall perennial herb, dry meadow, montane zone of the Swiss Alps. Expanding ray-like, vessel free zones separate radial strips of vessels which are surrounded by pervasive parenchyma. (B) Diffuse porous xylem with marginal, not lignified parenchymatous zones in root collar of *Gadellia lactiflora*, 80 cm tall perennial herb, meadow, subalpine zone of the Georgian Caucasus. Unlignified ray-like structures separate the radial vessel/fibre/parenchyma strips. (C) Diffuse porous xylem without fibres and tangential cracks (ring shakes) in the vessel-free, ray-like radial zone in root collar of *Campanula rotundifolia,* 10 cm tall perennial herb, meadow, montane zone of the Swiss Alps. Ring boundaries do not exist within the fibre/parenchyma zones. Primary vascular bundles keep their form over many years. Annual species with mandatory fibre formation, very small vessels, absent axial parenchyma and rays and different bark structures. (D) A large zone consisting of lignified fibres and vessels surrounds the pith. Dark spots in the large phloem represent collapsed sieve tubes in root collar of a *Campanula erinus*, 5 cm tall herb in a dry meadow of the Mediterranean zone, Crete, Greece 40x. Cells in the active cambium are not lignified (blue). (E) As (D) but with a small and not distinctly structured phloem. The cambium is no longer active. Root collar of a *Legousia falcata*, 20 cm tall herb, meadow of the hill zone in Switzerland. 40x. Perennial species with dense phellem belts. (F) Squeezed xylem due to high tensile strength of the phellem. The originally radial vessel/parenchyma strips are bent. Root collar of *Phyteuma orbiculare*, 20 cm tall herb, meadow, subalpine zone of the western Alps of France. Polarized light. 40x. (G) The phellem consists of rectangular cork cells, which are produced by an active phellogen. Root collar of *Campanula elatinoides*, 20 cm tall herb, Botanical garden Bern, hill zone of Switzerland.

**Figure 3:**
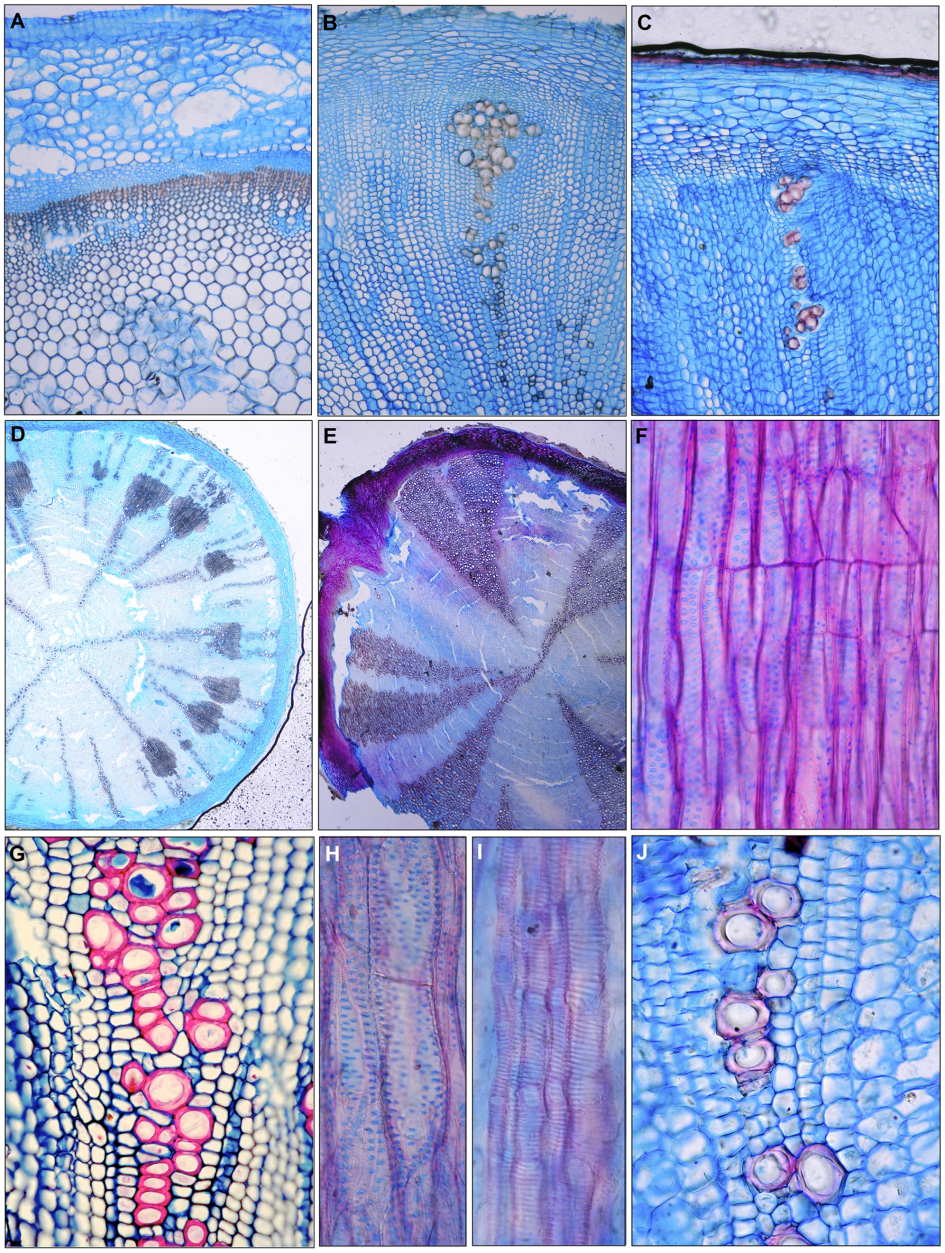
*Phyteuma ovatum*, 40 cm tall perennial herb, meadow of the subalpine zone in the Austrian Alps. (A) Pith, xylem and bark of a flower stalk. The xylem consists mainly of lignified fibres. 100x. (B) Pith, xylem and bark of the upper part of a polar root (root collar). The xylem consists mainly of unlignified parenchyma cells and radial arranged vessel groups. Fibres are absent. 100x. (C) Pith, xylem and bark of a bulb. The xylem consists mainly of unlignified parenchyma cells and small radially arranged vessel groups. Fibres are absent. Representative of a species with optional fibre formation. (D) Groups of fibres occuring in the third ring of root collar of *Campanula rotundifolia*, 20 cm tall, perennial herb, cultivated under optimal conditions, garden, Zürich, Switzerland. (E) Fibre-free stem in root collar of *Campanula rotundifolia*, 20 cm tall perennial herb, dry meadow, montane zone of the Swiss Alps. The radial wedges consist of parenchyma and vessels. (F) Scalariform perforations with 2 and 3 bars in root collar of a *Campanula drabifolia*, 5 cm tall perennial herb in a dry meadow of the Mediterranean zone, Crete, Greece. (G) Thick-walled vessels arranged in irregular groups in root collar of *Campanula glomerata*, 20 cm tall perennial herb, dry meadow, hill zone of Switzerland. (H) Round inter-vessel pits in a vessel within a fibre-zone. Root collar of *Campanula medium*, 40 cm tall perennial herb, meadow, Mediterranean zone of France. (I) Scalariform intervessel pits in vessels within a zone of pervasive parenchyma. Root collar of *Campanula medium*, 40 cm tall perennial herb, meadow, Mediterranean zone of France. 400x. (J) Thick-walled vessels within a thin-walled parenchyma zone of root collar of *Campanula medium*, 40 cm tall perennial herb, meadow, Mediterranean zone of France.

Transverse, tangential and radial sections were cut from a total of 122 individuals (see Figure 2 for examples). Since anatomical differences exist between roots, bulbs, root collars and annual flower stalks (Figure 3a–d), comparisons of anatomical sections were exclusively based on sections within the transition between the hypocotyl and the primary root (root collar). In this zone all annual rings of perennial plants do exist and the reaction to mechanical stress seems to be reduced to a minimum. All samples were stored in 40% ethanol before being sectioned with a sliding microtome. Sections were simultaneously stained with Safranin and Astrablue, dehydrated with ethanol and xylene, and mounted in Canada balsam [28]. The anatomical descriptions of the xylem are based on the IAWA List of microscopic features for hardwood identification [29] and specific xylem and phloem features of herbs based on Schweingruber et al. [30].

### Phylogenetic Analysis

All the sequences used in this study were obtained from genBank (www.ncbi.nlm.nih.gov). Our goal was to get maximum overlap with the pre-existing morphological dataset and yet not to clutter the alignment with excessive unknown positions. A combination of five loci satisfied this condition: internal transcribed spacer (ITS), trnT-trnL intergenic spacer, matK+trnK region, the gene for rubisco large subunit (rbcL) and petB-petD intergenic spacer. Hence the full-length ITS locus was not always available and sometimes a concatenate of ITS1+ ITS2 was used; all the accession numbers can be found in Table S2, available online. The L-INS-i algorithm implemented in the online version of MAFFT 6 [31] was employed to align the sequence datasets. Partial alignments were concatenated, manually adjusted in BioEdit [32] and subdued to the *automated1* algorithm in trimAll software [33] to exclude highly divergent and gap-rich regions.

Prior to the phylogenetic analysis, the best-fit model was selected by Kakusan4 [34], where the baseml software [35] served as the computational core and both non-partitioned and partitioned models were evaluated. According to the Bayesian information criterion [36], we finally used the GTR model with rate variation across sites simulated by discrete gamma distribution (Γ8), autocorrelated by the AdGamma rates prior and unlinked for particular gene partitions. To reflect the increased probability of transitions over transversions in non-coding loci, we set the substitution rates prior (revMatPr) for the ITS, trnT-L and petB-D partition to the Dirichlet function with values 1 and 3.

The phylogenetic analysis in itself was represented by the Bayesian inference (BI), conducted in MrBayes version 3.1.2 [37]. This comprised two independent runs with four Metropolis-coupled MCMC chains of 1×10^7^ generations sampled after every 1000th generation. In every run, one Markov chain was cold and three were incrementally heated by a parameter of 0.3. To eliminate trees sampled before reaching apparent stationarity, the first 25% of entries were discarded as burn-in and the rest was used to compute the majority-rule consensus (Figure 1).

The tree inferred by MrBayes served as groundwork for further evaluation of the anatomical trait dataset. All the anatomical traits were coded as binary data (49 traits in total) and confronted with the tree in Mesquite version 2.6 [38]. Various criteria of character fitting were observed and will be discussed later, from the retention index RI [39], to the tracing of character history. In the latter case, two unequal rates of transition between character states, depending on the direction of transition, were assumed (asymmetrical 2-parameter Markov k-state model) [40], and the branch lengths were taken into account (Figure 1).

### Relationships between Anatomy, Ecological Preferences and Phylogeny

Relationships between the anatomical structure of the studied species and their ecological preferences (habitat type, elevation) and competitive ability (expressed by plant height at the adult stage, which is an important trait in asymmetric competition for light) were evaluated using linear models, redundancy analysis RDA; see Legendre and Legendre [41], fitted for both (i) individual anatomical traits (single response variable model) and, (ii) all anatomical traits analyzed together as response variables in a global multivariate test. Type I errors were estimated using non-parametric Monte Carlo permutation tests, based on the *F* statistic, with 1999 random permutations. Since part of the explanatory power uncovered by relating the anatomical traits to ecological preferences might be alternatively explained by phylogenetic inertia affecting both the similarity of anatomical traits among closely related taxa and the similarity of ecological niches that such taxa occupy, we decided to use so-called phylogenetic corrections. The method of Diniz-Filho et al. [42], as modified by Desdevises et al. [11], was used. Here, the variation explained by the phylogenetic relatedness of species was removed from the model, using species coordinates on selected axes of a principal coordinate analysis (PCoA) calculated from a patristic distance matrix corresponding to the MrBayes phylogenetic tree described above.

Selected principal coordinates, which were used as covariates during tests including phylogenetic correction, were also used as predictors for individual anatomical traits to estimate the amount of variation in the trait values explained by species phylogeny [11]. Particular attention was paid to the relationships between the anatomical traits (selected based on the highest fit in the global RDA analysis) and plant height and elevation. All statistical methods were applied using Canoco 5 [43], including the estimation of linear models (performed using RDA with a single response variable). The family-wise error rate was accounted for by Bonferroni correction of significance values.

## Results

### Anatomical Structure

All of the analysed annual and perennial Campanulaceae had secondary radial growth in the root collar zone. Growth rings of varying distinctness occurred in most perennial species (Figure 2), but this varied within species and between individuals. For example, rings could be very distinct at the periphery of the stem but absent at the centre. Ring boundaries were expressed by semi-ring porosity (Figure 2a), marginal parenchyma (Figure 2b) or ring shake in the large rays (Figure 2c). The ages of plants with distinct rings varied. Four of the species were annual and therefore had only one ring, 15 species had 2 to 4 rings, ten species had 5–10 rings and ten species had 10 to 21 rings. Annual rings tended to be very narrow: 0.15 to 0.5 mm in 25 species, 0.55 to 0.9 mm in 7 species, and 1 to 2 mm in 7 species.

Two principal stem constructions occurred within the studied taxa (Figures 2 and 3). One group had no fibres (Figure 2 a–c) or, if present, fibre zones were sporadic. This group included all *Phyteuma* and many *Campanula* species. Species that lacked fibre in their stem centres may have had peripheral fibre zones. In the 12 species where this occurred, the central stem was classified as juvenile, while the peripheral section was classified as adult. This division is slightly problematic as fibre-formation within a species seems to relate to the precise position in the root collar zone (Figure 3 d–e).

The second group consisted of stems composed mainly of fibres and vessels (Figure 2 d–e), such as *Legousia sp.* and tall *Campanula spp*. Vessel diameters of all the measured species were small. Early wood vessels of plants under 20 cm in height had a diameter of 15–25 µm and those of larger plants 25–50 µm. The length of early wood vessels varied between 80 and 600 µm. Minimum vessel lengths averaged between 80 and 100 µm in small arctic and alpine plants (e.g., *Campanula giesekiana*, *Phyteuma globulariifolia*). Maximum vessel length averaged between 200 to 300 µm in the 5 cm tall *Campanula erinus*. Species with heights from 10 to 80 cm (N = 16) had vessel lengths between 100 and 200 µm. In species with large amounts of parenchyma (Figure 2 a–c) (*Phyteuma spp., Jasione montana*, alpine species such as *Campanula alpina, C. alpestris, C. barbata*), vessels tended to be solitary or grouped into short radial or irregular clusters (Figure 3a and 3g), whereas long radial multiples occurred in species with a large proportion of fibres (Figure 2a and 2f–g).

Perforation plates of all of the species were simple, with the exception being *Campanula drabifolia* and occasionally *Campanula erinus.* Among the many simple perforations a few plates had1 to 3 horizontal bars (Figure 3f). Two distinct types of inter-vessel pits occurred. Pits were round or slightly laterally enlarged in vessels of fibre-rich zones (as in most *Campanula spp.* from lower elevation meadows and dry mediterranean sites such as *Campanula glomerata, C. persicifolia, C. pelviformis, C. drabifolia,* but also *Legousia falcata*), and scalariform in areas with pervasive parenchyma (*Phyteuma spp., Jasione montana, and Campanula* spp. from alpine zone). Therefore, species with large, fibre-rich zones contained exclusively round pits and those without fibres contained only scalariform inter-vessel pits. Since some species contained fibre-rich and fibre-less zones, both pit types existed in the same individuals (Figure 3h–i). In addition, small thick-walled vessels were combined with the presence of pervasive parenchyma in the absence of fibres (Figure 2a–c and 3g).

Fibres were thin or thin-to-thick walled, and had small (<3 µm) oblique, slit-like pits without small pit borders (libriform fibres). Peripheral fibre belts occurred in the genera *Campanula, Petromarula* and *Jasione* but not in the genus *Phyteuma*.

**Figure 4:**
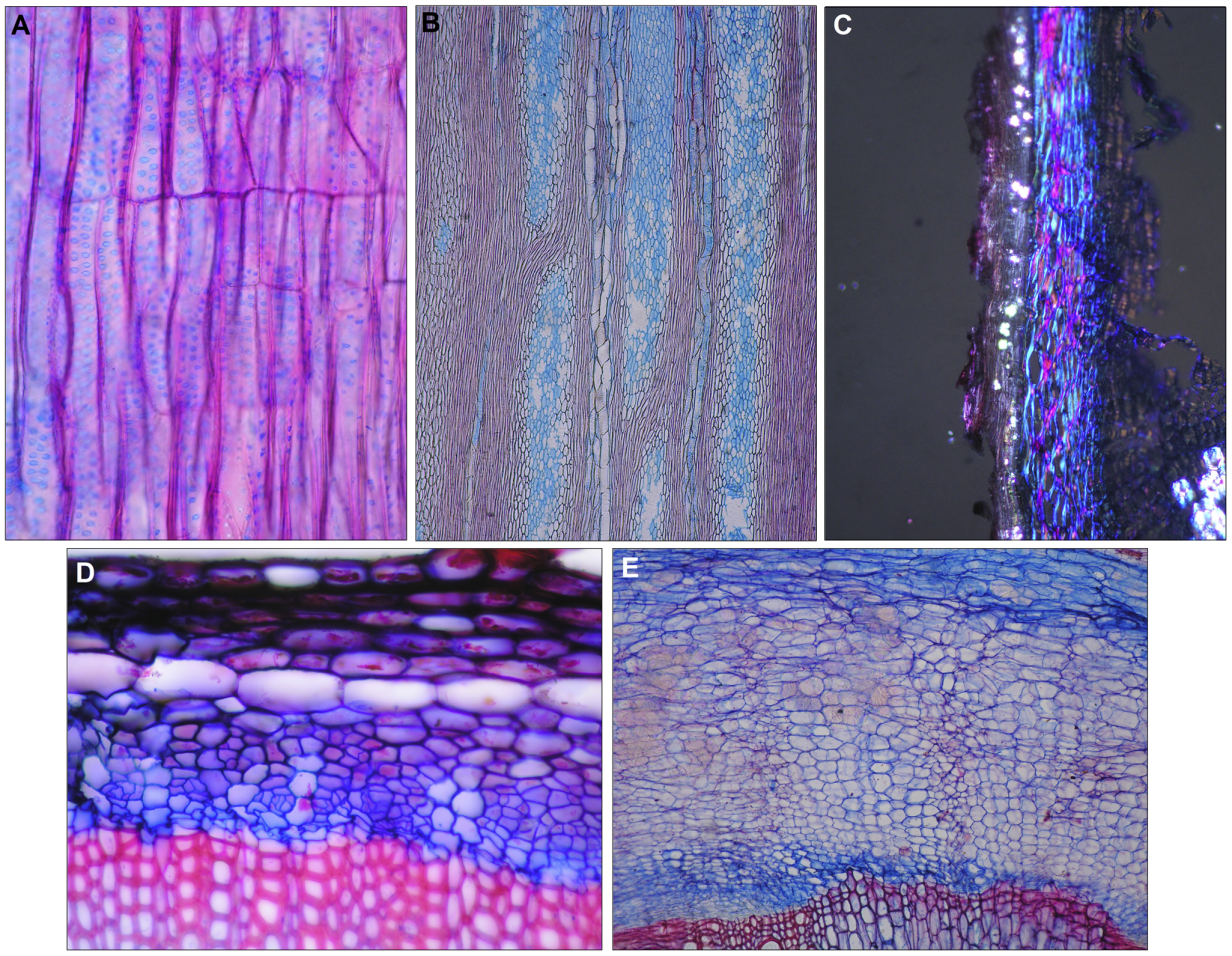
(A) Radial section of a uniseriate ray with upright cells. Root collar of a *Campanula drabifolia*, 5 cm tall perennial herb in a dry meadow of the Mediterranean zone, Crete, Greece. (B) Tangential section of very large rays, partially confluent with the axial tissue. Root collar of *Gadellia lactiflora*, 80 cm tall perennial herb, meadow, subalpine zone of the Georgian Caucasus. (C) Crystal sand in the phellem. Root collar of *Campanula giesekiana*, 5 cm tall perennial herb, on rock, arctic zone, Greenland. Polarized light. (D) Phloem with small groups of sieve tubes and companion cells. Root collar of *Campanula rapunculoides*, 40 cm tall perennial herb, meadow, hill zone of Switzerland. (E) Phloem with small groups of sieve tubes and companion cells between large parenchyma zones. Root collar of *Petromaerula pinnata*, 80 cm tall perennial herb, meadow, Mediterranean zone, Crete, Greece.

Axial parenchyma was mainly pervasive. In fibre-less zones the unlignified parenchyma cells were thin-walled and surrounded vessels (Figure 3g–i); parenchyma was mostly absent in fibre-rich zones (Figure 2d–e) and the only species containing vasicentric paratracheal parenchyma was *Gadellia lactiflora* (Figure 2b). Typical rays were rare in the present material. Uniseriate rays occurred in some individuals of species with fully lignifed stem centres (*Campanula erinus*) or large fibre belts (e.g., *C. drabifolia,* Figure 4b). Upright cells of uni-seriate rays appeared extremely small in tangential sections and multi-seriate rays occurred in *Gadellia lactiflora* (Figure 2b and 4a). Large parenchymatic zones between vascular bundles appeared as rays in cross-sections (Figure 2 and 3b,d), but they cannot be defined as rays in tangential sections. Rays within vessel-fibre strips were entirely absent.

The basic phloem-structure of all species consisted of parenchyma cells. The phloem included locally smaller and larger round or radial groups of sieve tubes and companion cells. They remained turgid for the lifespan of the plant (Figure 4d–e) or collapsed during the first growing season (Figure 2d). The phellem of many species formed a dense belt consisting of rectangular cork cells (Figure 2f), and its tensile force often exceeded the turgor of parenchymatous cells in the xylem. Therefore, the radially-organized xylem structure was deformed (Figure 2g), making ring counting and ring-width measurements problematic. Laticifers were absent or were anatomically not differentiated from other cells in the phloem and the cortex of the Campanuloideae. Finally, crystal sand occurred in all genera and most species, but only in the phellem (Figure 4c).

### Evolutionary Trends in Anatomical Traits

We included 39 Campanuloidae species in the phylogenetic evaluation - those for which anatomical traits were known together with at least one sequence of interest. In congruence with recent research [44]–[46], the genus *Campanula* was found to be deeply polyphyletic, forming two major clades accompanied by several somewhat isolated lineages (*C. uniflora*, *C. persicifolia* and *Campanula*/*Gadellia lactiflora*). In accordance with Wendling et al. [46], the clade involving *C. erinus* and *C. bononensis* (supported by the 1.00 Bayesian posterior probability) is flagged as *Campanula* s. str., the second large cluster (containing e.g. *C. rapunculus* and *C. scheuchzeri*; BPP = 1.00) can be identified with *Rapunculus* 1 clade and it groups firmly with *Adenophora pereskiifolia* (BPP = 1.00). *Campanula uniflora*, along with *Petromarula pinnata*, has strong adherence to the genus *Phyteuma* (“phyteumoid clade”, BPP = 0.98); together with *Legousia* spp. and possibly also *C. persicifolia*, this group (BPP = 1.00) matches clade *Rapunculus* 2 of Wendling et al. [46].

**Figure 5:**
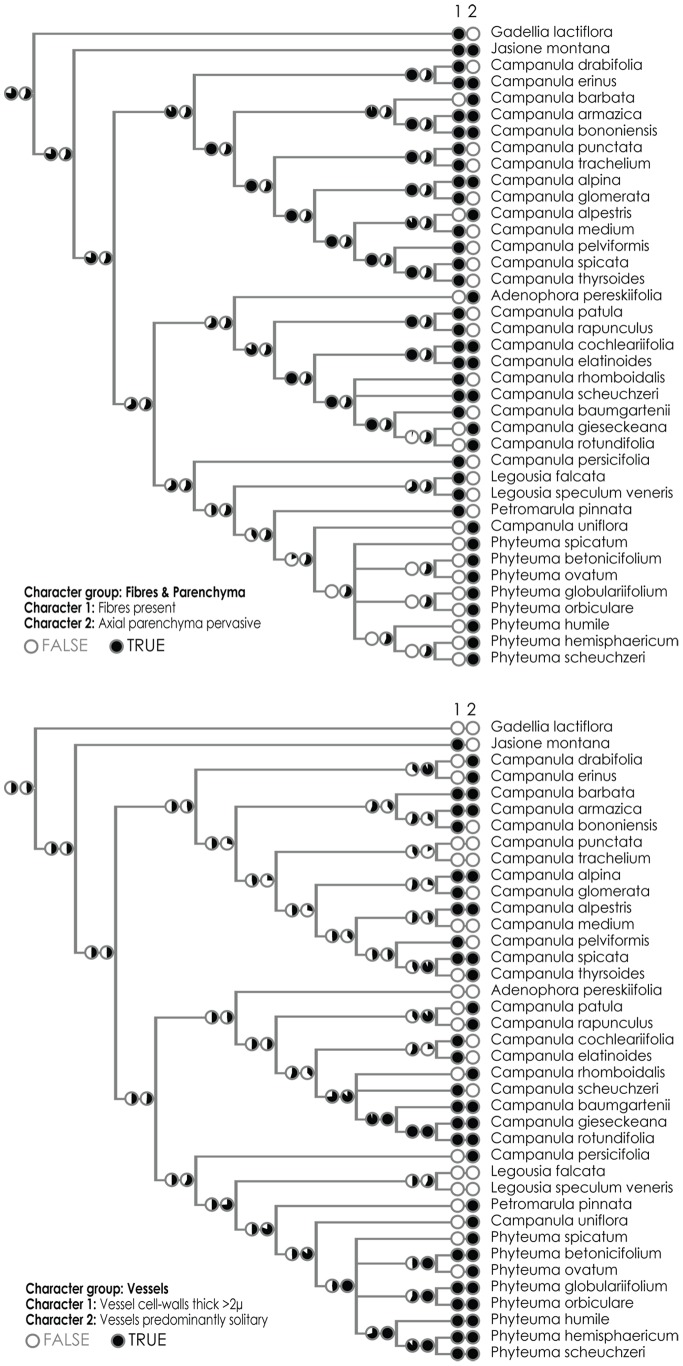
Maximum likelihood reconstruction of three best fitting characters: fibres present/absent (RI = 0.69), axial parenchyma pervasive (RI = 0.44), vessel cell-walls thick >2 µ (RI = 0.47), vessels predominantly solitary (RI = 0.31). The calculation was done in accordance with the asymmetrical 2-parameter Markov k-state model as implemented in Mesquite 2.6 (Maddison and Maddison, 2009).

**Figure 6:**
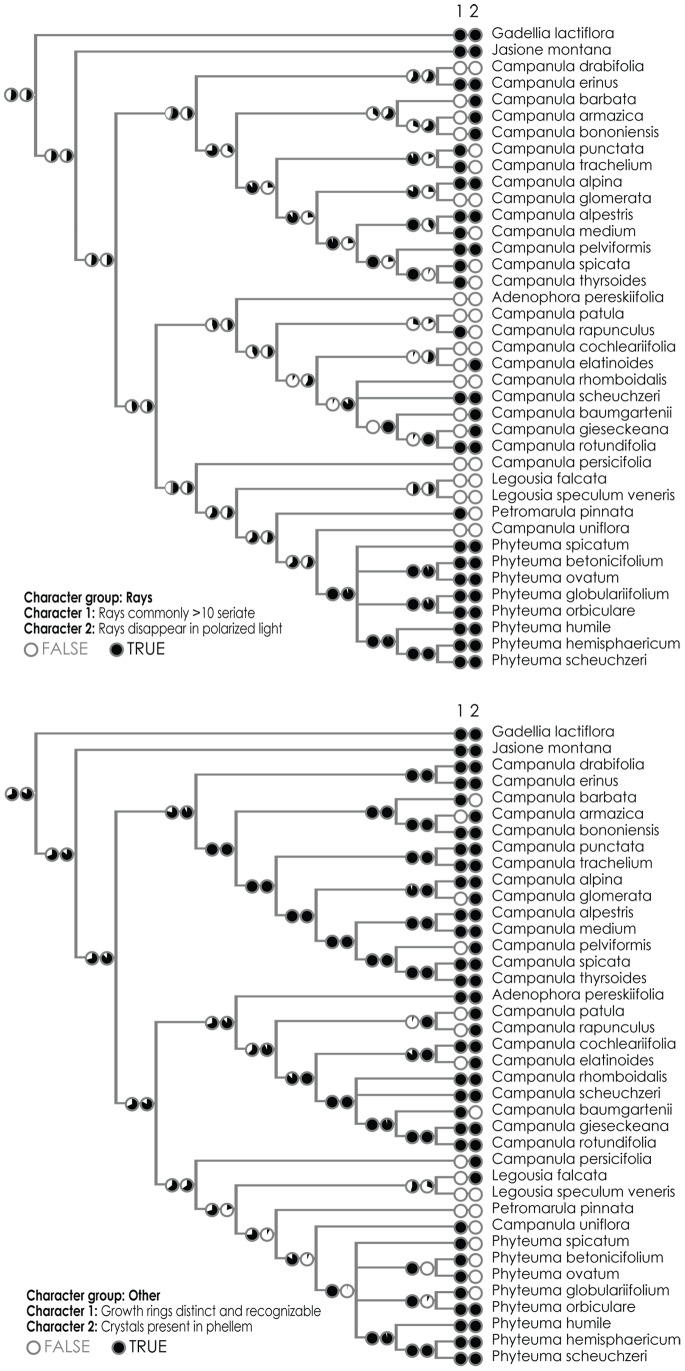
Maximum likelihood reconstruction of three best fitting characters: rays commonly >10 seriate (RI = 0.47), rays disappear in polarized light (RI = 0.44), growth rings distinct and recognizable (RI = 0.33), crystals present in phellem (RI = 0.38).

The dataset consisting of anatomical traits contained a very low phylogenetic signal (Retention Index is 0.343 for displayed tree) due to their highly plastic nature, both developmental and ecological. Yet, some evolutionary trends can be seen within the main lineages with the “phyteumoid” clade especially distinguished by quite a large number of characters (absence of fibres, pervasive parenchchyma, and type of rays). The reconstructions of ancestral states for several chosen characters are shown in Figures 5 and 6. Traits connected with the quality of fibres are among the most congruent with phylogeny and the retention index for several of them is above 0.5 (RI = 0.69 in the case of presence/absence of fibres); they firmly support the close relationship between *C. uniflora* and *Phyteuma spp.* Presence/absence of fibres is moreover the only character for which the ancestral state for the whole Campanuloidae subfamily can be unequivocally reconstructed under the decision threshold of 1.0 (Figure 5); the Proportional Likelihoods are 0.77∶0.23 in favour of their presence and all the cases of fibre loss should be therefore considered as secondary. Other well fitting characters can be found also amongst traits describing the morphology of vessels and rays (Figure 6), although the variability within major clades is quite high.

### Relationships between Plant Anatomical Traits, their Ecological Preferences, Adult Plant Height and Phylogeny for the Displayed Tree

**Figure 7:**
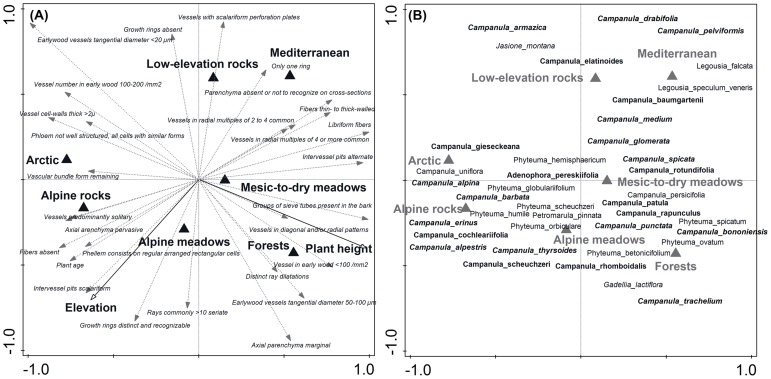
Ordination diagrams with the first two partial RDA axes (after accounting for phylogenetic relations among species using the methods of Desdevises *et al*. (2003)) showing how anatomical traits of plant species are related to their habitats, elevation and adult plant height. Arrows in the left diagram (A) point in the direction of increasing expected values of corresponding anatomical traits, symbols in the right diagram (B) represent individual plant species, and the triangles show the type of habitat (ecological niches) that individual taxa occupy. Relative values of individual species for a trait can be deduced by a perpendicular projection of the species symbols onto the trait arrow; the Pearson correlation coefficient is approximated by the cosine of the angle between the arrows of two traits being compared [40]. Different styles were used to separate the groups of species belonging to individual clades (bold italic for Campanula s. str., bold for Rapunculus 1, and normal for Rapunculus 2 clade).

The ecological attributes (habitat type, elevation) and adult plant height had a significant effect on the divergence of anatomical traits, and altogether explained 34.9% of the total variation in anatomical traits between species. This relationship remained significant after accounting for phylogenetic relatedness among taxa in the phylogeny-corrected analysis (*P*<0.01). The results of comparisons without phylogenetic corrections (hereafter ahistorical comparison) and phylogeny-corrected analysis showed similar patterns. Therefore, only results from the latter analysis are presented in the RDA ordination diagram (Figure 7). The main anatomical differences along the first (horizontal) ordination axis are associated with decreasing adult plant height from the arctic to temperate zones, i.e., the first axis separated taller plants such as *Campanula trachelium, C. persicifolia, C. rapunculus*, *Phyteuma spicatum* and *P. ovatum,* typical of temperate meadows and forests from smaller taxa such as *Campanula gieseckeana, C. cochleariifolia*, *C. uniflora*, *C. alpine* and *Phyteuma humile* which occupy a rocky alpine and arctic environment. The taller plants included distant taxa from all three evolutionary clades (*Campanula* s. str., *Rapunculus 1* and *Rapunculus* 2) that converged towards a similar anatomical structure characterized by large vessel tangential diameters of 50–100 µm, smaller numbers of vessels in early wood (<100/mm^2^), alternate intervessel pits and groups of sieve tubes in phloem. Smaller Arctic and alpine taxa, however, had more vessels in early wood (100–200/mm^2^), with smaller tangential diameter <20 µm and thicker vessel cell-walls >2 µm, pervasive axial parenchyma and poorly structured phloem with all cells with similar forms. The changes along the second axis seemed to be associated with habitat preferences along elevational gradients from dry and warm Mediterranean and temperate, open rocky sites to cool and moist alpine meadows and screes. The second axis separated species such as *Campanula pelviformis*, *C. baumgartenii*, *Jasione montana*, *C. elatinoides* and *Legousia falcata*, typical for the Mediterranean and lower elevation temperate habitats, from alpine taxa such as *Campanula scheuchzeri, C. alpestris*, *Phyteuma orbiculare* and *P. humile.* The anatomical features for these alpine meadow taxa included distinct growth rings, the absence of fibres, greater longevity (higher number of annual rings), scalariform intervessel pits, and phellem consisting of regularly arranged rectangular cells. Compared to the alpine species, those taxa typical for the dry and warm Mediterranean sites were shorter-lived, with thin- to thick-walled fibres present at the periphery of the stem but absent in the centre. Vessels were arranged in diagonal and/or radial patterns and were missing parenchyma.

Table 1 gives an overview of partial RDA analyses with a single response variable and single predictor, together with the fraction of variation in the measured anatomical traits that can be accounted for by evolutionary history, elevation and plant height. Stepwise selection of principal coordinates, representing components of phylogenetic relatedness that significantly explain differences in anatomical properties, indicated that five axes should be considered and these explained 27.2% of the total variation in all anatomical traits. When only plant age was analyzed as a single response variable, the evolutionary history accounted for 24.7% of the variation, the plant height 4.6% of the variation and elevations 16.3%. The significant negative relationship between plant age and height was lost after phylogenetic correction, while the positive relationship with elevation was retained. A significant negative relationship was found for the presence of fibres and plant height, with a positive relationship found between the presence of fibres and elevation. This effect manifested itself both in phylogeny-corrected and ahistorical (uncorrected) tests (*P*<0.05). The largest amount of variation explained by evolutionary history concerned vessel diameter and fibres.

**Table 1:** Results of partial tests of relationships between selected individual anatomical traits of plants (in rows) and their adult plant height and elevation.

		Plant height				Elevation			
Anatomical traits	Evol (%)	Res	AH	PC	Ecol (%)	Res	AH	PC	Ecol (%)
Plant age	24.7	−	0.03	ns	4.6	+	0.004	0.011	16.3
Vessel diameter <20 µm	21.9	−	0.000	0.001	39.9		ns	ns	0.1
Vessel diameter 20–50 µm	63.1		ns	ns	0.6		ns	ns	0.9
Vessel diameter 50–100 µm	26.7	+	0.02	0.045	8.1		ns	ns	0.06
Intervessel pits sclariform	39.2		ns	ns	0	+	0.001	0.003	24.4
Thick-walled vessels >2 µm	23.4	−	0.004	0.047	10.7		ns	ns	5.9
Fibres absent	29.1	−	0.007	0.012	17.3	+	0.002	0.03	13.5
Parenchyma pervasive	31.9	−	0.064	0.062	9.9	+	0.002	0.005	21.7
Vascular bundles remaining	56.1	−	0.013	0.024	15.4		ns	ns	0.1
Libriform fibres	11.2	+	0.007	0.002	20.9	−	0.009	0.002	18.5
Fibres absent in the stem centre	64.1		ns	ns	1.45		0.054	ns	4.8
Parenchyma absent	47.2	+	0.046	ns	6.46	−	0.01	0.006	18.5
Bark with groups of sieve tubes	25.2	+	ns	0.007	20.9		ns	ns	2.4
Phellem cells rectangular	35.8	−	0.053	ns	1.62	+	0.091	0.006	20.6
Crystals in phellem	33.6		ns	ns	0.12		ns	ns	4.6

AH headed columns refer to ahistorical comparisons (i.e without phylogenetic corrections), while PC headed columns refer to models with a correction for phylogenetic relatedness. Individual cells in AH and PC columns show Type I error probability estimates (adjusted by Bonferroni correction within test families) or ns when adjusted with P≥0.10. The percentage of trait variation explained by phylogenetic relatedness (Evol) between the species, and ecological preferences (Ecol) in the phylogeny-corrected analysis, are shown. Response (Res) columns show positive (+) or negative (−) relationships between the anatomic trait value and the predictor.

## Discussion

To our knowledge, this is one of the first studies where the anatomical features of so many species from wide-ranging habitats have been related to morphological and ecological traits and phylogenetic history data derived from DNA sequences. We acknowledge that the selection of species in a subfamily as large as Campanuloideae (the *Camplanula* genus itself includes over 500 species) is difficult. In this paper, the availability of material has been the basis for inclusion which means that some parts of the subfamily are overrepresented and some underrepresented. At the same time, however, the studied species do represent major clades of the subfamily. Both significant variability in anatomical traits and major habitats, from low-elevation Mediterranean to high-elevation alpine and Arctic areas, are well represented. We believe that carefully interpreted inference about species’ anatomical adaptation to the environment from a smaller, but nonetheless representative, set of common species covering a wide range of habitats and observed variability in traits can significantly contribute to the ecological understanding of the evolution of plant structures. This is particularly true when such inference is based on a modern phylogenetic tree constructed for target species from nucleotide sequences. We found no major conflict between previous phylogenetic works on Campanulaceae [14], [47] and our phylogeny. It is quite interesting to note that after ten years, using other methods and different data sources (loci), we achieved consonance with Eddie et al. [14]. We added several new species to the picture using a combination of five loci, also including those unlinked with chloroplast genes, which are countable as independent data sources.

Phylogenetic and ecological assessments of anatomical features within the Campanulaceae family have so far been limited by the fact that most species in the literature are tropical shrubs and trees belonging to the subfamily Lobelioideae [1]. However, in this study all the species described are annual or perennial herbs from seasonal temperate regions. Until now, most herbaceous species of Lobelioideae have not been characterized, thus comparisons of the present material is only possible with the well-documented material of Carlquist [1]. As a systematic study of herbaceous species of Lobelioideae is lacking, phylogenetic and ecologic interpretations for the entire family are still under debate.

Based on the findings in this study a number of features seem to be primarily related to phylogeny. The family is characterized by vessels with simple perforation plates. However, some herbs and shrubs of the Campanuloideae and Lobelioideae have subdivided types with one to three bars [1], [21]. Scalariform perforation plates do not separate any systematic groups. The occurrence of libriform fibres is not a reliable feature for separating subfamilies, as they form the basic tissue in all shrub and tree Lobelioideae, which also occurs within the Campanuloideae. The fibre-rich and parenchyma-lean tissue of the species *Legousia speculum-veneris, Petromarula pinnata* and some *Campanula spp.* separate them from all *Phyteuma* spp. (“Phyteumoid” clade) within the Campanuloideae, although septate fibres [1] occur only in the subfamily Lobelioideae. Distribution patterns of parenchyma cells are specific for subfamilies as well as for species. Parenchyma is absent in *Legousia*, in the fibre-rich zones of all Campanuloideae species and in the herb *Lobelia syphilitica.* Pervasive parenchyma occurs only in herbaceous Campanuloideae species. The presence of vasicentric parenchyma, which is specific for all shrub- and tree like Lobeliaceae [1], cannot be confirmed with the material prepared in this study.

The presence of rays and their structure appear to be of taxonomic value. Large, distinct rays separate Lobelioideae shrubs and trees, and the large herb *Gadellia lactiflora* from all other species at the very least. What appear as large rays in the xylem of herbaceous Campanuloideae actually represent vessel-free parenchymatous zones between vascular bundles and not true rays, and rays are not present within vascular bundles either. A characteristic for the Campanuloideae is the presence of crystal sand in the phellem. Limited stem-anatomical material, and the lack of consensus among various researchers on a classification below the Campanuloideae and Lobelioideae [12] prohibit further taxonomic conclusions.

A number of features that evolved repeatedly during evolution in ‘unrelated’ taxa from the Campanuloideae subfamily seem to be primarily related to habit adaptation and ecology. The taller, low-elevation plants from all tree evolutionary clades (*Campanula* s. str., *Rapunculus 1* and *Rapunculus* 2) tended to converge towards similar anatomical structure characterized by large vessel tangential diameter, smaller number of vessels in early wood and alternate intervessel pits. Small-stature alpine and Arctic taxa developed, on the other hand, distinct growth rings, an absence of fibres, greater longevity (higher number of annual rings), and scalariform intervessel pits. Formation of annual rings is a response to intra-annual weather and climate changes. In the present material, rings occured mostly in plants from highly seasonal climates compared to other Campanulaceae species from tropical zones. Explaining annual rings of small plants based on their growth habit and environmental factors is problematic because several stem components, and not only the xylem, optimize stems for water transport, stability and nutrition storage. Despite these restrictions, some relational observations can be made. Plant size determines ring width, where small plants have smaller rings than large plants and annual rings are generally smaller in plants found at high altitudes than those growing at low altitudes. However, since plant size is related to altitude, which is more than simply a temperature gradient effect, the dominant influencing factor remains unexplained. Interestingly, age trends occur in perennial herbs, as average ring width normally decreases with age, which is commonly observed in shrub and tree species.

### Conclusions

The large anatomical variability of the subfamily Campanuloideae found within the studied material was partitioned into phylogenetic relatedness and ecological adaptation. The main evolutionary trend concerns the separation of the “Phyteumoid” clade as distinguished by quite a large number of characters (absence of fibres, pervasive parenchyma). Despite clear phylogenetic inertia in the anatomical features studied, several important links between these traits and adult plant height and habitat preferences were found after removing the effects of phylogenetic relatedness. The observed variability in early wood vessel parameters (diameter, cell-wall thickness, number) seems to be best predicted by adult plant height (as a proxy of species competitiveness) as related to the gradient of productivity. This is represented in our study by species occurrence in habitats from mesic forests and meadows to alpine and Arctic rocky sites. Variability in plant age, parenchyma and fibre type is related to species optima along altitudinal gradients from low-elevation Mediterranean to high-elevation alpine meadows. The large anatomical variability of the family Campanulaceae found within the available material can mainly be explained through taxonomic differences between the subfamilies Lobelioideae and Campanuloideae. One of the questions that remains, however, is how many features are life form specific? Hence, it would be interesting to repeat such a comparative study with the focus on similar life forms from both subfamilies, not limiting the choice just to trees and shrubs [1]. We hope that our novel, comparative approach, which attempts to understand the evolution of plant anatomical structures through separation of evolutionary inertia from a true adaptation to the environment, will prompt new research in this area.

## Supporting Information

Table S1 **Summary table of plant species studied.**
(DOCX)

Table S2 **The accession numbers of nucleotide sequences (internal transcribed spacer (ITS), trnT-trnL intergenic spacer, matK+trnK region, the gene for rubisco large subunit (rbcL) and petB-petD intergenic spacer) obtained from GenBank (** www.ncbi.nlm.nih.gov/nuccore/ **).**
(DOCX)
