# Supplementary material for: Variation in Stem Anatomical Characteristics of Campanuloideae Species in Relation to Evolutionary History and Ecological Preferences
Source: PLoS One. 2014 Feb 21;9(2):e88199. doi: 10.1371/journal.pone.0088199 (PMC3931624; doi:10.1371/journal.pone.0088199)
Supplement: Table S1 — Summary table of plant species studied. (DOCX) [file pone.0088199.s001.docx]

**Table S1.**

| Species studied | Plant Height (cm) | Life form | Habitat | Altitude (m a.s.l.) | Latitude | Longitude | Locality | Country |
| --- | --- | --- | --- | --- | --- | --- | --- | --- |
| Adenophora pereskiifolia | 100 | H | Mixed deciduous forests | 1300 | 41 | 126 | Changbai | China |
| Campanula alpestris | 5 | H | Alpine rocks | 2500 | 46 | 6 | Col du Galibier Lautaret | France |
| Campanula alpina | 5 | H | Alpine rocks | 1600 | 46 | 14 | Masun, Lijubliana | Slovenia |
| Campanula armazica | 5 | H | Low-elevation rock | 800 | 43 | 45 | Botanical Garden Tbilisi | Georgia |
| Campanula barbata | 20 | H | Alpine meadows | 1400 | 46 | 8 | Gasterntal | Switzerland |
| Campanula baumgartenii | 40 | H | Low-elevation rock | 200 | 46 | 17 | Breitenbrunn, Burgenland | Austria |
| Campanula bononiensis | 80 | H | Low-elevation meadows | 800 | 46 | 14 | Kostnicke | Slovenia |
| Campanula cochleariifolia | 5 | H | Alpine rocks | 2200 | 47 | 10 | Scalotta, Lenzerheide | Switzerland |
| Campanula drabifolia | 5 | H | Mediterranean zone | 20 | 35 | 24 | Phaistos Crete | Greece |
| Campanula elatinoides | 20 | H | Low-elevation rock | 530 | 47 | 8 | Botanical Garden Bern | Switzerland |
| Campanula erinus | 5 | H | Alpine rocks | 2300 | 46 | 12 | Lienzer Dolomiten | Austria |
| Campanula gieseckeana | 5 | H | Arctic zone | 68 | 55 | -24 | Karupelv- Valley | Greenland |
| Campanula glomerata | 40 | H | Low-elevation meadows | 450 | 47 | 8 | Birmensdorf ZH | Switzerland |
| Gadellia lactiflora | 80 | H | Alpine meadows | 2200 | 42 | 44 | Tskara-Tskaro-Pass | Georgia |
| Campanula medium | 40 | H | Low-elevation rock | 900 | 44 | 6 | Castellane, Provence | France |
| Campanula patula | 40 | H | Low-elevation meadows | 550 | 47 | 16 | Rosaliengebirge, Vienna | Austria |
| Campanula pelviformis | 20 | T | Mediterranean zone | 300 | 35 | 24 | Agia Galini, Crete | Greece |
| Campanula persicifolia | 40 | H | Low-elevation meadows | 500 | 48 | 11 | Solnhofen Bavaria | Germany |
| Campanula punctata | 30 | H | Mixed deciduous forests | 740 | 41 | 126 | Changbai | China |
| Campanula rapunculus | 40 | H | Low-elevation meadows | 600 | 47 | 8 | Huttwil BE | Switzerland |
| Campanula rhomboidalis | 40 | H | Alpine meadows | 1600 | 46 | 7 | Piccolo San Bernardo | Italy |
| Campanula rotundifolia | 30 | H | Low-elevation meadows | 1600 | 45 | 6 | Névache, Briançon | France |
| Campanula scheuchzeri | 20 | H | Alpine meadows | 2300 | 46 | 10 | Faulhorn BE | Switzerland |
| Campanula spicata | 40 | H | Low-elevation meadows | 800 | 47 | 8 | Orsière VS | Switzerland |
| Campanula thyrsoides | 20 | H | Alpine meadows | 2300 | 46 | 6 | Mt. Tendre VD | Switzerland |
| Campanula trachelium | 80 | H | Mixed deciduous forests | 1050 | 46 | 13 | Turrach, Steiermark | Austria |
| Campanula uniflora | 10 | H | Arctic zone | 50 | 55 | -24 | Scorsbysund | Greenland |
| Jasione montana | 20 | H | Low-elevation meadows | 600 | 39 | -4 | Castilia | Spain |
| Legousia falcata | 20 | T | Mediterranean zone | 700 | 28 | -16 | Chio, Tenerife | Spain |
| Legousia speculum veneris | 20 | T | Mediterranean zone | 1000 | 44 | 6 | Embrun Var | France |
| Petromarula pinnata | 80 | H | Mediterranean zone | 100 | 35 | 24 | Plakias Crete | Greece |
| Phyteuma betonicifolium | 40 | H | Alpine meadows | 1600 | 46 | 8 | Mt.Bar TI | Switzerland |
| Phyteuma globulariifolium | 5 | H | Alpine meadows | 2400 | 46 | 12 | Grossglockner | Austria |
| Phyteuma hemisphaericum | 5 | H | Alpine meadows | 2000 | 46 | 8 | San Bernardino GR | Switzerland |
| Phyteuma humile | 5 | H | Alpine meadows | 3000 | 46 | 8 | Gornergrat, Zermatt VS | Switzerland |
| Phyteuma orbiculare | 20 | H | Alpine rocks | 1800 | 44 | 6 | Mt. Ventoux, Provence | France |
| Phyteuma ovatum | 40 | H | Alpine meadows | 1700 | 46 | 11 | Timmelsjoch-Pass | Austria |
| Phyteuma scheuchzeri | 20 | H | Alpine rocks | 1500 | 46 | 8 | Mt.Bar TI | Switzerland |
| Phyteuma spicatum | 40 | H | Mixed deciduous forests | 600 | 47 | 8 | Uetliberg, ZH | Switzerland |
