# Supplementary material for: Variation in Stem Anatomical Characteristics of Campanuloideae Species in Relation to Evolutionary History and Ecological Preferences
Source: PLoS One. 2014 Feb 21;9(2):e88199. doi: 10.1371/journal.pone.0088199 (PMC3931624; doi:10.1371/journal.pone.0088199)
Supplement: Table S2 — The accession numbers of nucleotide sequences (internal transcribed spacer (ITS), trnT-trnL intergenic spacer, matK+trnK region, the gene for rubisco large subunit (rbcL) and petB-petD intergenic spacer) obtained from GenBank ( www.ncbi.nlm.nih.gov/nuccore/ ). (DOCX) [file pone.0088199.s002.docx]

**Table S2.**

| petB-petD |  |  |  |  |  |  |
| --- | --- | --- | --- | --- | --- | --- |
| JX915238.1 | Adenophora | pereskiifolia | Adenophora_pereskiifolia |  |  |  |
| JX914669.1 | Adenophora | divaricata | Adenophora_divaricata |  |  |  |
| JX915201.1 | Campanula | alpestris | Campanula_alpestris |  |  |  |
| JX914819.1 | Campanula | alpina | Campanula_alpina |  |  |  |
| FN396988.1 | Campanula | armazica | Campanula_armazica |  |  |  |
| JX914966.1 | Campanula | barbata | Campanula_barbata |  |  |  |
| FN396991.1 | Campanula | baumgartenii | Campanula_baumgartenii |  |  |  |
| JX914998.1 | Campanula | tridentata | Campanula_tridentata |  |  |  |
| JX915200.1 | Campanula | bononiensis | Campanula_bononiensis |  |  |  |
| JX914738.1 | Campanula | cochleariifolia | Campanula_cochleariifolia |  |  |  |
| JX915174.1 | Campanula | drabifolia | Campanula_drabifolia |  |  |  |
| FN397008.1 | Campanula | elatinoides | Campanula_elatinoides |  |  |  |
| JX915206.1 | Campanula | erinus | Campanula_erinus |  |  |  |
| JX914748.1 | Campanula | gieseckeana | Campanula_gieseckeana |  |  |  |
| JX915156.1 | Campanula | glomerata | Campanula_glomerata |  |  |  |
| JX915131.1 | Campanula | lactiflora | Campanula_lactiflora |  |  |  |
| FN397024.1 | Campanula | medium | Campanula_medium |  |  |  |
| JX914974.1 | Campanula | patula | Campanula_patula |  |  |  |
| JX914785.1 | Campanula | pelviformis | Campanula_pelviformis |  |  |  |
| JX915226.1 | Campanula | persicifolia | Campanula_persicifolia |  |  |  |
| JX915031.1 | Campanula | punctata | Campanula_punctata |  |  |  |
| JX914708.1 | Campanula | rapunculus | Campanula_rapunculus |  |  |  |
| FN397033.1 | Campanula | rhomboidalis | Campanula_rhomboidalis |  |  |  |
| JX915164.1 | Campanula | rotundifolia | Campanula_rotundifolia |  |  |  |
| JX915162.1 | Campanula | scheuchzeri | Campanula_scheuchzeri |  |  |  |
| FN397044.1 | Campanula | spicata | Campanula_spicata |  |  |  |
| FN397047.1 | Campanula | thyrsoides | Campanula_thyrsoides |  |  |  |
| JX914678.1 | Campanula | trachelium | Campanula_trachelium |  |  |  |
| JX915039.1 | Campanula | uniflora | Campanula_uniflora |  |  |  |
| JX915216.1 | Jasione | montana | Jasione_montana |  |  |  |
| JX914808.1 | Legousia | falcata | Legousia_falcata |  |  |  |
| FN397071.1 | Legousia | speculum-veneris | Legousia_speculum-veneris |  |  |  |
| JX915212.1 | Petromarula | pinnata | Petromarula_pinnata |  |  |  |
| FN397084.1 | Phyteuma | betonicifolium | Phyteuma_betonicifolium |  |  |  |
| FN397085.1 | Phyteuma | hemisphaericum | Phyteuma_hemisphaericum |  |  |  |
| JX915214.1 | Phyteuma | humile | Phyteuma_humile |  |  |  |
| JX915213.1 | Phyteuma | ovatum | Phyteuma_ovatum |  |  |  |
| FN397086.1 | Phyteuma | scheuchzeri | Phyteuma_scheuchzeri |  |  |  |
| FN397080.1 | Pentaphragma | sp | Pentaphragma_sp |  |  |  |
| rbcL |  |  | _ |  |  |  |
| KC146507.1 | Adenophora | divaricata | Adenophora_divaricata |  |  |  |
| EU713381.1 | Campanula | bononiensis | Campanula_bononiensis |  |  |  |
| FJ587247.1 | Campanula | cochleariifolia | Campanula_cochleariifolia |  |  |  |
| FJ587252.1 | Campanula | drabifolia | Campanula_drabifolia |  |  |  |
| EU713398.1 | Campanula | erinus | Campanula_erinus |  |  |  |
| KC146541.1 | Campanula | glomerata | Campanula_glomerata |  |  |  |
| EU643703.1 | Gadellia | lactiflora | Gadellia_lactiflora |  |  |  |
| FJ587261.1 | Campanula | medium | Campanula_medium |  |  |  |
| JN893008.1 | Campanula | patula | Campanula_patula |  |  |  |
| EU713350.1 | Campanula | pelviformis | Campanula_pelviformis |  |  |  |
| FJ587264.1 | Campanula | persicifolia | Campanula_persicifolia |  |  |  |
| KC146539.1 | Campanula | punctata | Campanula_punctata |  |  |  |
| FJ587272.1 | Campanula | rapunculus | Campanula_rapunculus |  |  |  |
| FJ587273.1 | Campanula | rotundifolia | Campanula_rotundifolia |  |  |  |
| JN571984.1 | Campanula | scheuchzeri | Campanula_scheuchzeri |  |  |  |
| FJ587281.1 | Campanula | spicata | Campanula_spicata |  |  |  |
| EU643723.1 | Campanula | thyrsoides | Campanula_thyrsoides |  |  |  |
| FJ587285.1 | Campanula | trachelium | Campanula_trachelium |  |  |  |
| JN571986.1 | Campanula | uniflora | Campanula_uniflora |  |  |  |
| Y08462.1 | Campanula | viburnea | Campanula_viburnea |  |  |  |
| EU713354.1 | Jasione | montana | Jasione_montana |  |  |  |
| EU713418.1 | Legousia | falcata | Legousia_falcata |  |  |  |
| EU713365.1 | Legousia | speculum-veneris | Legousia_speculum-veneris |  |  |  |
| AJ419699.1 | Pentaphragma | ellipticum | Pentaphragma_ellipticum |  |  |  |
| EU713433.1 | Petromarula | pinnata | Petromarula_pinnata |  |  |  |
| EU643712.1 | Phyteuma | spicatum | Phyteuma_spicatum |  |  |  |
| trnL-trnF |  |  | _ |  |  |  |
| HQ704665.1 | Adenophora | divaricata | Adenophora_divaricata |  |  |  |
| EF213141.1 | Campanula | alpestris | Campanula_alpestris |  |  |  |
| EF213160.1 | Campanula | tridentata | Campanula_tridentata |  |  |  |
| JX445917.1 | Campanula | cochleariifolia | Campanula_cochleariifolia |  |  |  |
| EF088719.1 | Campanula | drabifolia | Campanula_drabifolia |  |  |  |
| FJ426578.1 | Campanula | elatinoides | Campanula_elatinoides |  |  |  |
| EF088720.1 | Campanula | erinus | Campanula_erinus |  |  |  |
| HQ704671.1 | Campanula | glomerata | Campanula_glomerata |  |  |  |
| FJ589212.1 | Gadellia | lactiflora | Gadellia_lactiflora |  |  |  |
| HQ823490.1 | Campanula | medium | Campanula_medium |  |  |  |
| EF213148.1 | Campanula | patula | Campanula_patula |  |  |  |
| EF088743.1 | Campanula | persicifolia | Campanula_persicifolia |  |  |  |
| HQ704667.1 | Campanula | punctata | Campanula_punctata |  |  |  |
| EF088758.1 | Campanula | rapunculus | Campanula_rapunculus |  |  |  |
| EF088759.1 | Campanula | rotundifolia | Campanula_rotundifolia |  |  |  |
| EF088762.1 | Campanula | scheuchzeri | Campanula_scheuchzeri |  |  |  |
| EF088769.1 | Campanula | spicata | Campanula_spicata |  |  |  |
| EF088774.1 | Campanula | trachelium | Campanula_trachelium |  |  |  |
| FJ426574.1 | Campanula | uniflora | Campanula_uniflora |  |  |  |
| GQ984071.1 | Cuttsia | viburnea | Cuttsia_viburnea |  |  |  |
| JX445918.1 | Jasione | montana | Jasione_montana |  |  |  |
| EF088786.1 | Petromarula | pinnata | Petromarula_pinnata |  |  |  |
| JX445920.1 | Phyteuma | spicatum | Phyteuma_spicatum |  |  |  |
| matK |  |  | _ |  |  |  |
| KC146495.1 | Adenophora | divaricata | Adenophora_divaricata |  |  |  |
| JN571946.1 | Campanula | cochleariifolia | Campanula_cochleariifolia |  |  |  |
| EU713291.1 | Campanula | erinus | Campanula_erinus |  |  |  |
| KC146501.1 | Campanula | glomerata | Campanula_glomerata |  |  |  |
| EU713318.1 | Gadellia | lactiflora | Gadellia_lactiflora |  |  |  |
| EU713272.1 | Campanula | medium | Campanula_medium |  |  |  |
| JN895650.1 | Campanula | patula | Campanula_patula |  |  |  |
| EU713243.1 | Campanula | pelviformis | Campanula_pelviformis |  |  |  |
| EU713324.1 | Campanula | persicifolia | Campanula_persicifolia |  |  |  |
| KC146499.1 | Campanula | punctata | Campanula_punctata |  |  |  |
| HE970684.1 | Campanula | rapunculus | Campanula_rapunculus |  |  |  |
| JN571959.1 | Campanula | rotundifolia | Campanula_rotundifolia |  |  |  |
| JN571962.1 | Campanula | scheuchzeri | Campanula_scheuchzeri |  |  |  |
| HE970685.1 | Campanula | trachelium | Campanula_trachelium |  |  |  |
| JN571964.1 | Campanula | uniflora | Campanula_uniflora |  |  |  |
| GQ983640.1 | Cuttsia | viburnea | Cuttsia_viburnea |  |  |  |
| EU713247.1 | Jasione | montana | Jasione_montana |  |  |  |
| EU713311.1 | Legousia | falcata | Legousia_falcata |  |  |  |
| EU713258.1 | Legousia | speculum-veneris | Legousia_speculum-veneris |  |  |  |
| AJ429387.1 | Pentaphragma | ellipticum | Pentaphragma_ellipticum |  |  |  |
| EU713326.1 | Petromarula | pinnata | Petromarula_pinnata |  |  |  |
| EU713254.1 | Phyteuma | spicatum | Phyteuma_spicatum |  |  |  |
| ITSs |  |  |  |  |  |  |
| HQ704521.1 | Adenophora | divaricata | all | Adenophora_divaricata_all | | |
| DQ304573.1 | Campanula | alpina | all | Campanula_alpina_all | | |
| AY322009.1 | Campanula | armazica | 1 | Campanula_armazica_1 | | |
| AY331422.1 | Campanula | armazica | 2 | Campanula_armazica_2 | | |
| AY322011.1 | Campanula | barbata | 1 | Campanula_barbata_1 | | |
| AY331424.1 | Campanula | barbata | 2 | Campanula_barbata_2 | | |
| DQ304619.1 | Campanula | beckiana | all | Campanula_beckiana_all | | |
| AY322043.1 | Campanula | tridentata | 1 | Campanula_tridentata_1 | | |
| AY331456.1 | Campanula | tridentata | 2 | Campanula_tridentata_2 | | |
| DQ304571.1 | Campanula | bononiensis | all | Campanula_bononiensis_all | | |
| DQ304578.1 | Campanula | drabifolia | all | Campanula_drabifolia_all | | |
| DQ304625.1 | Campanula | elatinoides | all | Campanula_elatinoides_all | | |
| DQ304580.1 | Campanula | erinus | all | Campanula_erinus_all | | |
| HQ704534.1 | Campanula | glomerata | all | Campanula_glomerata_all | | |
| EU177776.1 | Gadellia | lactiflora | all | Gadellia_lactiflora_all | | |
| HQ823432.1 | Campanula | medium | all | Campanula_medium_all | | |
| FM212739.1 | Campanula | patula | all | Campanula_patula_all | | |
| DQ304590.1 | Campanula | persicifolia | all | Campanula_persicifolia_all | | |
| HQ704547.1 | Campanula | punctata | all | Campanula_punctata_all | | |
| FM212738.1 | Campanula | rapunculus | all | Campanula_rapunculus_all | | |
| JN571988.1 | Campanula | rotundifolia | all | Campanula_rotundifolia_all | | |
| DQ304614.1 | Campanula | scheuchzeri | all | Campanula_scheuchzeri_all | | |
| DQ304574.1 | Campanula | spicata | all | Campanula_spicata_all | | |
| DQ304575.1 | Campanula | thyrsoides | all | Campanula_thyrsoides_all | | |
| DQ304572.1 | Campanula | trachelium | all | Campanula_trachelium_all | | |
| DQ304588.1 | Campanula | uniflora | all | Campanula_uniflora_all | | |
| DQ499092.1 | Cuttsia | viburnea | all | Cuttsia_viburnea_all | |  |
| DQ304566.1 | Jasione | montana | all | Jasione_montana_all | |  |
| DQ304589.1 | Legousia | falcata | all | Legousia_falcata_all | |  |
| AY322065.1 | Legousia | speculum-veneris | 1 | Legousia_speculum-veneris_1 | | |
| AY331478.1 | Legousia | speculum-veneris | 2 | Legousia_speculum-veneris_2 | | |
| DQ304582.1 | Petromarula | pinnata | all | Petromarula_pinnata_all | | |
| DQ304583.1 | Phyteuma | globulariifolium | all | Phyteuma_globulariifolium_all | | |
| AY322071.1 | Phyteuma | orbiculare | 1 | Phyteuma_orbiculare_1 | | |
| AY331484.1 | Phyteuma | orbiculare | 2 | Phyteuma_orbiculare_2 | | |
| DQ304584.1 | Phyteuma | spicatum | all | Phyteuma_spicatum_all | | |
